# Prevalence of G2P[4] and G12P[6] Rotavirus, Bangladesh

**DOI:** 10.3201/eid1301.060910

**Published:** 2007-01

**Authors:** Mustafizur Rahman, Rasheda Sultana, Giasuddin Ahmed, Sharifun Nahar, Zahid M. Hassan, Farjana Saiada, Goutam Podder, Abu S. G. Faruque, A. K. Siddique, David A. Sack, Jelle Matthijnssens, Marc Van Ranst, Tasnim Azim

**Affiliations:** * International Centre for Diarrhoeal Disease Research, Bangladesh, Dhaka, Bangladesh; † University of Leuven, Leuven, Belgium

**Keywords:** Group A rotavirus, diarrhea, genotype, G12P[6], G2P[4], Bangladesh, research

## Abstract

Approximately 20,000 stool specimens from patients with diarrhea visiting an urban and a rural hospital in Bangladesh during January 2001–May 2006 were tested for group A rotavirus antigen, and 4,712 (24.0%) were positive. G and P genotyping was performed on a subset of 10% of the positive samples (n = 471). During the 2001–2005 rotavirus seasons, G1P[8] (36.4%) and G9P[8] (27.7%) were the dominant strains, but G2[4] and G12P[6] were present in 15.4% and 3.1% of the rotavirus-positive patients, respectively. During the 2005–06 rotavirus season, G2P[4] (43.2%) appeared as the most prevalent strain, and G12P[6] became a more prevalent strain (11.1%) during this season. Because recently licensed rotavirus vaccines are specific for only P[8], these vaccines may not perform well in settings where non-P[8] types are prevalent.

## Abstract 2

Rotavirus strains not covered by licensed vaccines are increasing.

Group A rotaviruses are the major etiologic agents of severe infantile diarrhea. Worldwide, >125 million infants and young children develop rotavirus-associated diarrhea each year, resulting in 440,000 infant and child deaths, mostly in developing countries (*1*). In Bangladesh, rotaviruses cause 6,000–14,000 deaths each year in children <5 years of age (*2*).

The tremendous incidence of rotavirus disease underscores the urgent need for interventions such as vaccines, particularly to prevent childhood deaths in developing nations. Fortunately, 2 rotavirus vaccines, RotaTeq (Merck and Co., Whitehouse Station, NJ, USA) and RotaRix (GlaxoSmithKline, Research Triangle Park, NC, USA) passed a large safety trial, showed high efficacy against the major rotavirus G types, and have been approved by the Food and Drug Administration (*3*,*4*). The efficacy trials of these vaccines have been conducted in the United States, Latin America, and Europe but not in developing countries in Africa and Asia.

Rotavirus infection shows a characteristic seasonal pattern that is not clearly understood. In developed countries with temperate climates, peak incidence is in winter; however, in developing countries with tropical or subtropical climates, the virus circulates year-round (*5*–*8*). The temperature in Bangladesh is usually high from April through October and relatively low from December through February. In addition, previous studies have indicated that rotavirus in Bangladesh is affected by floods, which increase opportunities for transmission of the virus (*8*,*9*). Bangladesh lies on the confluence of hundreds of rivers and is inundated with water every year due to enhanced rainfall during the monsoon season, starting in June (*10*).

Rotaviruses belong to the genus *Reoviridae* and consist of 11 segments of double-stranded RNA. Two outer capsid proteins, VP7 (defining G genotypes) and VP4 (defining P genotypes), independently elicit neutralizing responses. Based on these proteins, a dual classification system of group A rotaviruses has been introduced (*5*). Rotaviruses can be serotyped by using neutralization assays with panels of antisera and genotyped by type-specific primer-dependent reverse transcription–PCR (RT-PCR) and nucleotide sequence analysis (*11*–*13*). So far, >15 G and 26 P genotypes have been described in humans and a variety of animals (*6*,*14*,*15*). The major human G types are G1, G2, G3, G4, and G9, which, combined with the P types P[8], P[4], and P[6], account for >80% of rotavirus-associated gastroenteritis episodes worldwide (*16*,*17*).

Rotaviruses show great genomic diversity, and several studies in different regions of Bangladesh have identified types not targeted by candidate rotavirus vaccines (*18*–*21*). Unicomb et al. (*8*) showed that frequent genomic reassortment among different rotavirus types was accelerated by mixed infection and generated huge genomic diversity. Although the importance of type-specific immunologic protection against rotavirus disease is still under discussion, many investigators suggest that genomic characterization of rotaviruses is needed to assess whether vaccine efficacy might be altered by the changing pattern in the distribution of different G and P genotypes (*17*,*22*,*23*).

In our study, stool samples from gastroenteritis patients admitted to 2 hospitals, 1 urban and 1 rural, within the hospital surveillance system of International Centre for Diarrhoeal Disease Research, Bangladesh (ICDDR,B), from January 2001 through May 2006 were tested for the rotavirus VP6 antigen. The study objective was to clarify the genomic diversity of rotavirus in urban and rural areas in Bangladesh, with a goal of providing information for rotavirus vaccine development programs.

## Materials and Methods

### Study Population

The ICDDR,B Centre for Health and Population Research runs an urban hospital situated in Dhaka, the capital city of Bangladesh, which has a population of ≈10 million, and a rural hospital at Matlab, 45 km southeast of Dhaka, which has ≈300,000 inhabitants. Each year, >100,000 patients are treated for diarrhea at the Dhaka hospital and ≈15,000 at the Matlab hospital. At the Dhaka hospital, diarrhea surveillance is conducted in a systematic manner; stool samples are collected to determine the presence of enteric pathogens in every 50th (2%) patient attending the hospital for treatment of diarrhea. In Matlab, hospital stool samples are collected from all patients from the community, which is under active rotavirus surveillance.

### Rotavirus Antigen Detection

As part of the surveillance system, rotavirus antigens (group A rotavirus-specific VP6 proteins) were detected in the stool specimens using a solid-phase sandwich-type enzyme immunoassay modeled after the Dakopatts commercial kit (Dakopatts, Copenhagen, Denmark), incorporating rabbit hyperimmune antisera produced at ICDDR,B and an anti-human rotavirus–horseradish peroxidase conjugate. The same criteria as those used by the Dakopatts kit were used for determination of positivity (*8*).

### RNA Extraction

Rotavirus RNA was extracted from the stool samples. The QIAamp Viral RNA mini kit (Qiagen/Westburg, Leusden, the Netherlands) was used according to the manufacturer’s instructions

### RT-PCR

A multiplex RT-PCR was performed by using the Qiagen OneStep RT-PCR Kit (Qiagen/Westburg) for rotavirus G and P genotypes using type-specific oligonucleotide primers as previously described (Table 1) (*11*,*13*,*24*). The reaction was carried out with an initial reverse-transcription step at 45°C for 30 min, followed by 35 cycles of amplification (30 sec at 94°C, 30 sec at 48°C, 1 min at 72°C), and a final extension of 7 min at 72°C in a thermal cycler (Eppendorf, Hamburg, Germany). PCR products were subjected to electrophoresis on a 2% agarose gel, stained with ethidium bromide, and observed under ultraviolet light. Specific segment sizes for different G and P genotypes were observed on the stained gel.

**Table 1:** Oligonucleotide primers used in the study for PCR amplification

Primer	Type	Position (nt)	Strand	Sequence (5′–3′)	Reference
Beg9	VP7	1–28	Plus	GGCTTTAAAAGAGAGAATTTCCGTCTGG	(*13*)
End9	VP7	1062–1036	Minus	GGTCACATCATACAATTCTAATCTAAG	(*13*)
RVG9	VP7	1062–1044	Minus	GGTCACATCATACAATTCT	(*13*)
Con2	VP4	868–887	Minus	ATTTCGGACCATTTATAACC	(*11*)
Con3	VP4	11–32	Plus	TGGCTTCGCCATTTTATAGACA	(*11*)
MR-G1	G1	314–335	Plus	CAAGTACTCAAATCAGTGATGG	Present study
MR-G2	G2	436–459	Plus	CTATGAATCCACAACTGTATTGTG	Present study
aET3	G3	689–709	Plus	CGTTTGAAGAAGTTGCAACAG	(*13*)
MR-G4	G4	480–499	Plus	GCTTCTGGTGAAGAGTTG	Present study
aAT8	G8	178–198	Plus	GTCACACCATTTGTAAATTCG	(*13*)
MR-G9	G9	757–776	Plus	GAACCATAAACTTGATGTG	Present study
MR-P8	P[8]	314–335	Minus	TCTACTGGATCGACGTGC	Present study
MR-P4	P[4]	474–494	Minus	CTATTATTAGAGGTTAAAGTC	Present study
3T-1	P[6]	259–278	Minus	TGTTGATTAGTTGGATTCAA	(*11*)
4T-1	P[9]	385–402	Minus	TGAGACATGCAATTGGAC	(*11*)
ND2	P[11]	116–133	Minus	AGCGAACTCACCAATCTG	(*11*)

### Nucleotide Sequencing

The PCR products were purified with the QIAquick PCR purification kit (Qiagen/Westburg) and sequenced by using the dideoxy-nucleotide chain termination method with the ABI PRISM BigDye Terminator Cycle Sequencing Reaction kit (Applied Biosystems, Foster City, CA, USA) on an automated sequencer. The consensus forward primer Beg9 and reverse primer End9 were used to amplify and sequence the VP7 gene. For the VP4 gene, the forward primer Con3 and reverse primer Con2 were used as described previously (*15*).

### Data Analysis and DNA Sequence Submission

Data were analyzed by SPSS for Windows, release 11.5.1 (SPSS Inc., Chicago, IL, USA). The nucleotide sequence data of the rotavirus strains were submitted to the GenBank under the accession nos. DQ482712, DQ482718, DQ482725, DQ146652, DQ146653, DQ146654, DQ146658, DQ146663, DQ146664, DQ146665, DQ146669, EF033338, EF033339, and EF033340.

## Results

### Detection of Rotavirus Antigen

From January 2001 through May 2006, 19,039 stool specimens were tested for group A rotavirus VP6 antigen; 4,644 (24.4%) samples had positive results. Table 2 shows the distribution of rotavirus-positive patients in the hospital surveillance systems in Dhaka and Matlab. The average detection rate of rotavirus was 25.2% in Dhaka and 23.3% in Matlab.

**Table 2:** Distribution of specimens positive for rotavirus, Bangladesh, January 2001–May 2006

Rotavirus season*	Dhaka		Matlab	
	No. tested	Rotavirus-positive (%)	No. tested	Rotavirus-positive (%)
2000–01†	879	214 (24.3)	715	202 (28.3)
2001–02	1,824	563 (30.9)	1,665	428 (25.7)
2002–03	1,806	458 (25.4)	1,583	338 (21.4)
2003–04	1,786	458 (25.6)	1,425	281 (19.7)
2004–05	2,374	521 (21.9)	1,547	350 (22.6)
2005–06	2,070	492 (23.8)	1,365	339 (24.8)
Total	10,739	2,706 (25.2)	8,300	1,938 (23.3)

*Each season starts in June and ends in May of the following year. †Data from January–May 2001 only.

### Quality Control

Stool specimens obtained from 311 patients with diarrhea were tested for the presence of rotavirus particles using the IDEIA rotavirus kit (DAKO Ltd., Cambridgeshire, UK). By using the IDEIA kit, 234 samples were found to be positive and 77 negative. By comparison, our in-house ELISA kit could detect rotavirus antigen in 232 of the IDEIA-positive samples. Among the IDEIA-negative samples, 72 were negative for rotavirus antigen by our in-house kit. Thus, a comparison of the results indicated that our in-house ELISA kit had an overall sensitivity of 99.1% and specificity of 96.1% compared with the IDEIA rotavirus kit.

### Age of the Rotavirus-positive Patients

**Figure 1:**
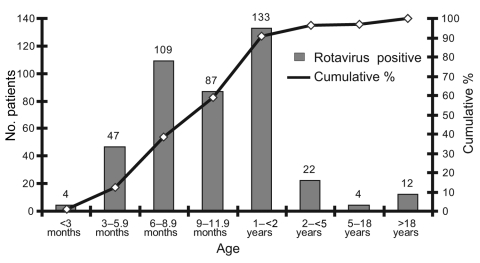
Age distribution for rotavirus-positive patients, Bangladesh, 2001–2005.

The age range of the rotavirus diarrhea patients (2001–2005) was 1 month–63.2 years, median age 10 months, and mean age 22.8 months. Most of the rotavirus-positive patients (91%) were <2 years of age (Figure 1). Infection rates were lowest in patients <3 months and >5 years of age.

### Seasonal Pattern of Rotavirus Infection

**Figure 2:**
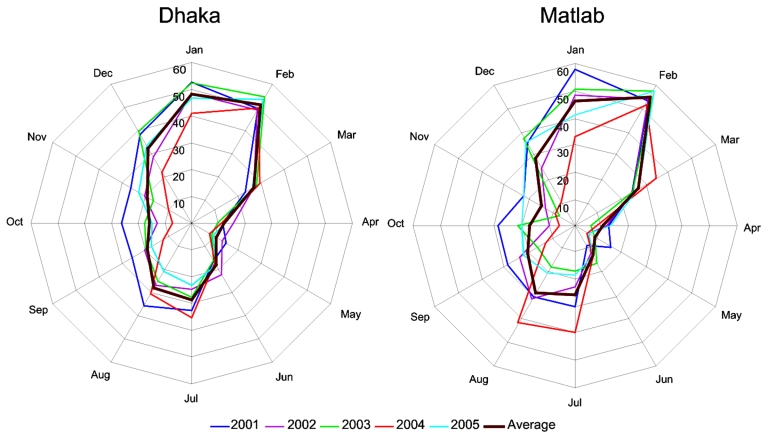
Distribution of rotavirus-positive patients by month, Dhaka and Matlab, Bangladesh. Percentages of positive rotavirus patients were calculated based on all diarrhea patients admitted to the Dhaka and Matlab hospital surveillance system during 2001–2005. The years are shown with different colored lines. The thick brown line represents the average for all years.

Figure 2 shows the monthly distribution of rotavirus diarrhea in Dhaka and Matlab. Rotaviruses were detected throughout the year in both settings, even though 2 clear seasonal rotavirus peaks were observed each year: a sharp winter peak in January and February, and a monsoon peak in July and August. Taking the average for each setting into account, our model suggests that the rotavirus season in Bangladesh usually starts in June and ends in May (year-round).

**Figure 3:**
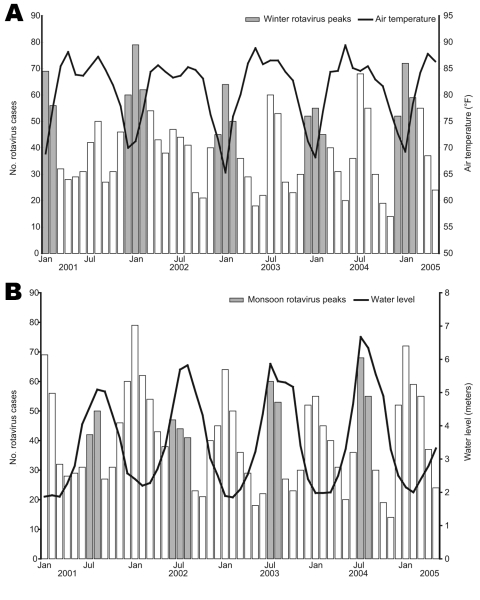
Correlation between cases of rotavirus diarrhea and air temperature and water level in Dhaka, Bangladesh, January 2001–May 2005.

Air temperature records for Dhaka and water level data for the Buriganga River (Sadarghat point, Dhaka) for the 5 years of the study (2001–2005) were obtained from the Institute of Water Modelling, Dhaka, Bangladesh (www.iwmbd.org). These data and the number of rotavirus patients admitted to the Dhaka hospital by month are shown in Figure 3. The temperature was lowest from December through February each year, which coincided with the increased number of rotavirus diarrhea cases. On the other hand, the monsoon peaks of rotavirus diarrhea were correlated with the water level. The water level reached the highest mark during July–August each year, which corresponded to the increase of the proportion of rotavirus diarrhea. The meteorologic data from Matlab also correlated to the increased incidence of rotavirus infection, as was seen for Dhaka (data not shown).

### Distribution of G and P Types

G and P genotyping were carried out on 471 rotavirus antigen–positive stool samples (10% of all rotavirus-positive patients) by using a type-specific primer–based multiplex RT-PCR that could detect 6 G genotypes (G1, G2, G3, G4, G8, G9) and 5 P genotypes (P[8], P[4], P[6], P[9], P[11]). The untypeable and suspicious samples with lower amounts of PCR products were successfully typed and confirmed by using nucleotide sequencing. Table 3 shows the distribution of G and P types of rotavirus strains detected in Dhaka and Matlab. No significant difference was observed between the distribution of rotavirus strains in Dhaka and Matlab (p>0.05). Overall, the most prevalent genotype was G1P[8] (33.8%), which was followed by G9P[8] (25.3%), G2P[4] (20.2%), and G4P[8] (8.3%). Mixed infections were detected in 3.2% of the samples. Strains with unusual G-P combinations, such as G1P[6], G2P[6], and G2P[8], were also detected.

**Table 3:** Distribution of G and P genotypes of rotavirus strains, Bangladesh, January 2001–May 2006

G type	P type	No. (%) rotavirus strains*		Total no. (%) rotavirus strains
		Dhaka	Matlab	
G1	P[6]	1 (0.4)	2 (1.0)	3 (0.6)
G1	P[8]	85 (31.3)	74 (37.2)	159 (33.8)
G2	P[4]	55 (20.2)	40 (20.1)	95 (20.2)
G2	P[6]	1 (0.4)	0	1 (0.2)
G2	P[8]	2 (0.7)	0	2 (0.4)
G4	P[8]	26 (9.6)	13 (6.5)	39 (8.3)
G9	P[6]	7 (2.6)	2 (1.0)	9 (1.9)
G9	P[8]	67 (24.6)	52 (26.1)	119 (25.3)
G11	P[6]	1 (0.4)	0	1 (0.2)
G11	P[8]	1 (0.4)	1 (0.5)	2 (0.4)
G12	P[6]	16 (5.9)	5 (2.5)	21 (4.5)
G12	P[8]	2 (0.7)	3 (1.5)	5 (1.1)
Mixed G/P		8 (3.0)	7 (3.5)	15 (3.2)
Total		272 (100.1)	199 (99.9)	471 (100.0)

*The percentage of the total for Dhaka is >100% and for Matlab <100% because each number was rounded off to the nearest one tenth of 1%.

Unusual porcine-like G11 rotavirus strains were detected in 3 patients (0.6%). These strains were untypeable by multiplex PCR because no G11-specific primer was included in the routine primer set. Therefore, sequencing of the VP7 and VP4 genes was required. The partial VP7 gene sequences of the 3 G11 rotavirus strains (Dhaka22-01, Matlab36-02, and Dhaka13-06) were most similar (>98% similarities at the nucleotide and >97% at the amino acid level) to the porcine-like G11 strain Dhaka6 (15). On the other hand, the VP4 genes were most similar to human P[8] or P[6] strains (Malawi strain OP351, Thai strain 15vp4w, and US strain Se585).

Uncommon human G12 rotavirus strains (5.6%), were also detected during our study period. Because the G12 strains were untypeable by using our routine primers, nucleotide sequencing of their VP7 genes was required. All the VP7 gene sequences of the Bangladeshi G12 strains were most similar to the recently isolated G12 strains (Indian strain ISO-2) but distantly related to the prototype G12 strain L26 isolated in the Philippines. The gene segments encoding the VP4, VP6, and NSP4 proteins were sequenced for Bangladeshi G12 strains Dhaka25-02 (G12P[8]) and Dhaka12-03 (G12P[6]). The VP4 gene sequence of strain Dhaka25-02 was most similar to the human P[8] rotavirus strain DRC88 (98% similarity at the amino acid level) isolated in Democratic Republic of the Congo, and strain Dhaka12-03 was most similar to the human P[6] strain US1205 (99% similarity at the amino acid level) isolated in the United States. The VP6 and NSP4 sequences of both strains were also most similar to human rotavirus strains (Indian rotavirus strains RMC100, G25795, and V13520).

Polyacrylamide gel electrophoresis was performed for the 26 G12 strains isolated in our study, and 18 showed a clear RNA migration pattern. Long electropherotypes were detected in 15 (83.3%) samples, which included both G12P[8] and G12P[6] strains. Short electropherotypes were detected in only 3 (16.7%) samples, which also included both G12P[8] and G12P[6] strains.

### Fluctuation of the G and P Types Distribution over Time

**Figure 4:**
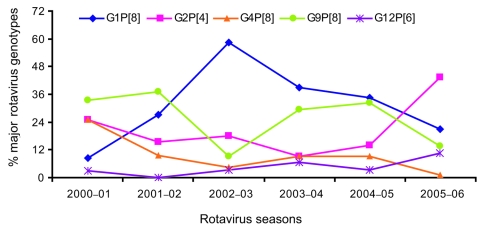
Temporal changes of the distribution of major rotavirus genotypes in Bangladesh, 2001–2006.

Large fluctuations of the rotavirus genotype distribution were observed both in Dhaka and Matlab. However, no significant difference was observed between the urban and rural setting with regard to the yearly distribution of genotypes (p>0.05). The overall distribution of the major genotypes over time is shown in Figure 4. The G1P[8] strains were less common in 2001, became the most predominant strains in the following years, but decreased again in 2005–06. G9P[8] strains dominated in the first 2 rotavirus seasons, decreased sharply during 2002–03, dominated again for the next 2 years, and decreased again during 2005–06. G4P[8], which had been the most prevalent strain in the 1990s in Bangladesh, was found to be less common in our study and constituted only 1.2% during 2005–06. Most interestingly, the strain G2P[4] was the most predominant (43.2%) during the 2005–06 rotavirus season, although it was less common during the previous seasons (15.4% 2001–05). The uncommon strains G12P[6] and G12P[8], introduced in Bangladesh for the first time during the 2000–01 season, became more prevalent (13.6%) in this region by the 2005–06 season.

## Discussion

Rotavirus was found in approximately one fourth of all patients with diarrhea who were treated at ICDDR,B hospitals; most rotavirus cases (92.5%) occurred in children during their first 2 years of life. A recent report indicated that 33% of children <5 years age admitted to the ICDDR,B from 1993 through 2004 were rotavirus positive (*2*). Reports from other Asian countries also indicated that rotaviruses were present in 20%–58% of patients with diarrhea who were <5 years of age. Thus, the rotavirus detection rate from our study is comparable to rates from some other Asian countries, including India, South Korea, and Hong Kong (20%–30%), but much lower than those reported in Taiwan, Thailand, China, Japan, Mayanmar, and Vietnam (43%–58%) (*25*–*34*).

Although rotavirus-associated diarrhea was documented year-round in Dhaka and Matlab, a sharp winter peak and a monsoon peak were observed each year. The winter rotavirus peak is usually observed worldwide, but the monsoon peak is not common in settings with temperate climates. We analyzed environmental data including rainfall and water level of the nearest river and found that the monsoon rotavirus peaks in Bangladesh could be defined by high water levels due to heavy rainfall, which normally starts in the second week of June (*35*). Because of the heavy rainfall, the water level of the rivers begins to increase and reaches its highest level during July–August each year, resulting in inundation of the surrounding areas and increasing the chance of fecal contamination of water. Ahmed and colleagues reported that the number of rotavirus diarrhea cases increased remarkably, and mixed rotavirus types were frequently isolated during the floods in 1988 in Dhaka (*10*). In July and August 2004 in Matlab (Figure 2), a large increase in rotavirus-associated diarrhea was observed. Analysis of the water level of the nearest river (Chandpur point of the Meghna River, data not shown) showed that this increase correlated directly with an increased water level. The water level reached the 5.42 meter mark in July 2004, the highest in that region during our study period.

Our main goal was to characterize the VP7 (G genotype) and VP4 (P genotype) gene segments of the rotavirus strains. We identified most of the globally common rotavirus types (G1, G2, G4, and G9) in our study. Surprisingly, no G3 strain has been detected in Bangladesh since 1993, even though G3 is one of the most prevalent rotavirus types worldwide (*8*,*25*,*29*). Results of rotavirus diversity from this study were compared with previous findings in Bangladesh (*8*), and we observed that the distribution of rotavirus genotypes was changing over time. From 1992 through 1997, the most common rotavirus genotype was G4 (47% of the typeable rotavirus strains), but this genotype’s prevalence gradually decreased, and it became a less common rotavirus strain over time (1.2% in 2005–06). The distribution of G2 strains, on the other hand, remained nearly unchanged through rotavirus season 2004–05 (19.5% in 1992–1997 and 16.2% in 2001–2005). However, G2 suddenly became the most prevalent genotype in 2005–06 (43.2%).

Three G11 strains, commonly found in pigs, were isolated from humans in the present study. In Bangladesh, pigs are uncommon farm animals, and no genotyping studies on pigs or other animals have been conducted. Therefore, the identification of the strains with an animal-like G11 VP7 specificity and a human P[8] or P[6] specificity raises the question whether these strains are reassortants of human and animal rotavirus strains. This finding underscores the need to include animal rotavirus strains rotavirus surveillance programs. At the same time, water samples, particularly those collected during floods, can be evaluated for the presence of unusual rotavirus strains that might have been introduced from domestic animals.

For the first time in Bangladesh, a very uncommon human rotavirus strain, G12, was detected. The strain was first detected in 1987–1988 in the Philippines, and since then, it has been emerging all over the world (*36*–*40*). G12 is reported as an important rotavirus strain in India (17.1% in 2003–2005) and in Argentina (6% in 1999–2003) (*17*,*36*). A considerable proportion of G12 was also documented during our study period and reached 13.6% in the latest rotavirus season (2005–06). Thus, the emergence of G12 strains has led to the need for prospective surveillance using new diagnostic RT-PCR primers for G12 strains.

Genetic analysis of the VP4, VP6, and NSP4 gene segments showed that the Bangladeshi G12 strains contained typical human rotavirus gene segments distantly related to the prototype G12 strains L26 and T152. It is possible that the VP7 gene segments from the prototype G12 strains were reassorted with the typical human rotavirus strains. More genetic analyses of complete genome sequences would be helpful to investigate the possible reassortment events and evolution of the recently emerging G12 strains.

P genotype analysis showed that the rotavirus strains with the P[8] specificity made up 76.4% of the circulating strains during 2001–2005; non-P[8] strains constituted 21.9%. The most interesting finding about P types in our study was that the non-P[8] strains represented more than half of the strains (56.8%) during the rotavirus season 2005–06. The currently licensed rotavirus vaccines have shown high efficacy rates in trials and have focused on the role of the major G genotypes, but the role of P genotypes has not been addressed clearly (*3*,*4*). These vaccines include the P[8] specificity, but it is unknown how the vaccines will perform in settings where the non-P[8] types are prevalent. An efficacy trial of the rotavirus vaccine RotaTeq will begin soon in Bangladesh, so the findings of our study regarding rotavirus strain diversity will be important for evaluating the results of this trial.
